# National burden of disabilities in Bangladesh and its socio-demographic correlates: A 2021 cross-sectional analysis

**DOI:** 10.1371/journal.pgph.0007463

**Published:** 2026-10-08

**Authors:** Mizanur Rahman, Md Shohel Rana, Gulam Khandaker, Md Mostafizur Rahman, Md Nuruzzaman Khan

**Affiliations:** 1 Department of Fisheries, Jamalpur Science & Technology University, Jamalpur, Bangladesh; 2 Department of Population Science, Jatiya Kabi Kazi Nazrul Islam University, Mymensingh, Bangladesh; 3 Research and Evaluation Unit, SAJIDA Foundation, Dhaka, Bangladesh; 4 CSF Global, Dhaka, Bangladesh; 5 Central Queensland Public Health Unit, Central Queensland Hospital and Health Service, Rockhampton, Australia; 6 School of Health, Medical and Applied Sciences, Central Queensland University, Rockhampton, Australia; 7 Department of Population Science and Human Resource Development, University of Rajshahi, Rajshahi, Bangladesh; 8 Nossal Institute for Global Health, Melbourne School of Population and Global Health, The University of Melbourne, Melbourne, Australia; VUMC: Vanderbilt University Medical Center, UNITED STATES OF AMERICA

## Abstract

Disability remains an important public health and social challenge, particularly in low- and middle-income countries (LMICs), including Bangladesh. However, nationally representative evidence on its burden, distribution, and associated factors remains limited. This study estimated the national prevalence and patterns of disability in Bangladesh and examined their sociodemographic correlates. This cross-sectional study analysed data from 155,025 participants in the 2021 National Survey on Persons with Disabilities (NSPD). Disability status (yes or no) was the primary outcome. Explanatory variables were measured at the individual, household, and community levels. Multilevel mixed-effects logistic regression models, stratified by age group (0–17 years [children] and 18–95 years [adults]), were used to examine factors associated with disability. The prevalence of disability was 2.79% (95% CI 2.69–2.91). Physical disability (42.44%) and visual impairment (14.11%) were the most commonly reported types, and 11.60% of persons with disabilities reported multiple disabilities. Overall, disability was more prevalent among males, although sex-specific patterns differed by age. Among children, girls had 31% higher adjusted odds of disability than boys (adjusted odds ratio [aOR] 1.31, 95% CI 1.13–1.52), whereas no significant sex was observed among adults (aOR 1.06, 95% CI 0.95–1.19). In both age groups, the odds of disability increased with years of schooling and household wealth. Among adults, housewives, students, and blue-collar workers had higher odds of disability than agriculture workers. Higher community-level literacy was associated with higher odds of disability among adults, whereas the association was reversed among children. The findings support strengthening community-based disability identification and referral in Bangladesh through existing primary health-care services, particularly in underserved populations and areas. Coordination between the Ministry of Health and Family Welfare and the Department of Social Services could improve access to existing social protection, rehabilitation, and assistive-device services.

## Introduction

Persons with disabilities (PWD) constitute the world’s largest minority group, with an estimated population of approximately 1.3 billion people globally, representing nearly 16% of the world’s population as of 2022 [1]. Over 80% of them reside in low- and middle-income countries (LMICs), which are defined as having a Gross National Income ranging between $1,146 and $4,515 per capita [2–4]. Notably, these figures are increasing rapidly worldwide, particularly within LMICs, driven by population ageing, improved survival from previously fatal conditions, epidemiological transition toward chronic diseases, and rising prevalence of disabilities associated with longer life expectancy [5,6]. Furthermore, LMICs are currently witnessing rapid urbanization and industrialization, leading to a growing workforce in manufacturing, urban informal jobs, and construction sectors, often lacking adequate occupational safety protections [7,8]. This situation has contributed to a substantial rise in accidents and injuries, accompanied by increasing mortality and long-term disability [9,10]. Additionally, road-traffic injuries are becoming increasingly prevalent in LMICs, contributing to approximately 1.35 million global deaths annually, with over 90% occurring in LMICs, while nearly 50 million additional individuals sustain non-fatal injuries, many of whom acquire permanent disability as a consequence [11–13].

Beyond these injury-related challenges, LMICs also face a substantial burden of disabilities arising from congenital and early-life conditions, including birth complications, neonatal infections, malnutrition, untreated childhood illnesses, and chronic conditions that often remain undiagnosed because of limited access to timely and quality healthcare [5,14]. Stroke is another critical, and often overlooked, contributor to disability burden: globally it ranked as the fourth leading cause of disability-adjusted life years (DALYs) in 2021, while in Bangladesh it is currently the third leading cause of death, underscoring its contribution to the country’s overall disability burden [15,16]. These factors are major contributors to neurodevelopmental disabilities such as intellectual disability, cerebral palsy, autism spectrum disorder, and learning difficulties, which together account for a substantial proportion of the global disability burden. Globally, an estimated 52.9 million children under five years live with developmental disabilities, with more than 95% residing in LMICs, reflecting both higher exposure to risk factors and substantial underdiagnosis in these settings [17,18]. Taken together, disability now represents one of the leading contributors to years lived with disability globally, placing significant pressure on healthcare systems, social protection programmes, and economic productivity, particularly in resource-constrained settings.

Bangladesh, a lower-middle-income country with a Gross National Income (GNI) of $2,064 and a Gross Domestic Product (GDP) per capita of approximately $2,854, represents an important context within this global trend [2,3]. It is the eighth most populous country in the world, with a population approaching 168 million [19]. The country is undergoing rapid urbanization and industrialization, consequently witnessing the simultaneous rise of road-traffic injuries and occupational hazards as increasingly common public health challenges [7,20,21]. These burdens are compounded by persistently high poverty levels and widespread engagement in hazardous occupations, including garment factories, construction work, transport services, rickshaw pulling, street-based employment, and informal daily labour, all of which substantially elevate injury-related disability risks [22–24]. The country also continues to struggle with ensuring continuous, high-quality maternal healthcare services capable of identifying and preventing adverse birth outcomes, including preterm birth and birth complications that frequently result in lifelong disability [25,26]. Beyond maternal care, inadequate post-stroke management, delayed diagnosis and treatment of vision impairment, cancer, and chronic diseases further contribute to long-term disability burden. Importantly, disability in Bangladesh creates substantial economic consequences, including reduced workforce participation, loss of household income, increased out-of-pocket expenditure for long-term treatment and rehabilitation, and increased dependency on family caregiving, collectively reinforcing cycles of poverty and social exclusion [16].

Persons with disabilities require substantial social and economic support for sustaining their livelihoods, particularly in LMICs, where they frequently experience marginalisation even within their own households and remain highly dependent on social assistance [27,28]. The highly competitive labour market, coupled with inadequate inclusive education systems, inaccessible communication systems, and poor transportation infrastructure, further exacerbates their vulnerability and dependency [29–31]. Together, these factors contribute to poorer educational outcomes, reduced labour force participation, poorer health outcomes characterised by higher mortality and multiple comorbidities, and reduced social participation due to stigma, exclusion, and restricted mobility [5,32]. Recognising these challenges, disability has increasingly become a major global development priority. International frameworks including the World Health Organization Global Disability Action Plan, the United Nations Convention on the Rights of Persons with Disabilities (CRPD), and the Sustainable Development Goals (SDGs) have all emphasised disability inclusion as central to achieving equitable development. Bangladesh has similarly introduced several national initiatives, including the Rights and Protection of Persons with Disability Act 2013, disability allowance programmes, and community-based rehabilitation efforts aimed at improving inclusion and reducing disability-related inequities [19,33]. However, effective implementation of these initiatives requires robust and nationally representative evidence on the true burden and distribution of disability.

Despite growing policy attention, research evidence from LMICs, including Bangladesh, remains limited and often fails to provide sufficiently robust evidence to support policymakers and programme planners in designing evidence-based interventions [34,35]. Country-level studies in LMICs, as well as global evidence more broadly, frequently fail to capture the true prevalence and multidimensional nature of disability, resulting in an incomplete understanding of its patterns and implications [1,36]. Furthermore, available evidence often produces contradictory findings because studies use different disability measurement approaches, rely on relatively small sample sizes, and frequently apply less rigorous analytical methods [37,38]. These limitations are particularly relevant in Bangladesh, where existing studies have reported highly inconsistent estimates of disability prevalence, making it difficult for policymakers to determine the true scale of the problem and effectively prioritise interventions [5,39]. More importantly, there remains limited nationally representative evidence examining how disability burden varies across socio-demographic groups, despite the fact that identifying these disparities is essential for targeting interventions and ensuring disability inclusion within broader national development agendas. As experiences of disability are highly context-specific and vary substantially across countries [40], these inconsistencies create significant challenges for policymakers and programme implementers in Bangladesh. Although global and national policy frameworks increasingly recognise disability as a development priority, effective implementation remains difficult without reliable population-level evidence identifying the groups most affected and the inequalities driving disability burden. Moreover, because the determinants of disability differ substantially between childhood, where congenital and early-life conditions predominate, and adulthood, where occupational, socioeconomic, and ageing-related pathways become more important, evidence that pools all ages together risks obscuring age-specific patterns that are directly relevant to designing early-childhood versus adult disability services. To address these evidence gaps, this study used data from the 2021 National Survey on Persons with Disabilities, the first dedicated, nationally representative disability survey conducted in Bangladesh, with two specific objectives: (i) to estimate the national-level burden and distribution of different types of disability, and (ii) to separately examine the individual-, household-, and community-level correlates of disability among children (0–17 years) and adults (18–95 years) in Bangladesh.

## Methods

### Sampling strategy

Data were obtained from the 2021 National Survey on Persons with Disabilities (NSPD) conducted by the Bangladesh Bureau of Statistics (BBS). The survey employed a two-stage stratified random sampling design to select study participants. In the first stage, 800 primary sampling units (PSUs) were randomly selected from the 293,579 PSUs listed in the 2011 Bangladesh National Population Census. In the second stage, 45 households were systematically selected from each sampled PSU. This sampling approach generated a target sample of 36,000 households, of which data were successfully collected from 35,493 households, corresponding to a response rate of 99.9%. The survey included all usual residents of participating households and collected data from 155,025 individuals, including 14,659 children aged 0–4 years, 39,513 children aged 5–17 years, and 100,853 adults aged 18–95 years. Further details of the sampling design and survey procedures are available in the survey report [41].

The NSPD was a dedicated, nationally representative disability survey specifically designed to estimate the prevalence, types, and correlates of disability in Bangladesh, rather than incorporating disability as a secondary module within a broader household survey. Data collection was conducted by trained field staff under the supervision of BBS following standardised survey protocols. As part of the survey quality assurance process, BBS implemented field supervision, double-entry verification, consistency checks, and range validation procedures to ensure data accuracy and completeness. The dataset used in the present study represents the final cleaned and de-identified version released by BBS following completion of these quality assurance procedures.

### Outcome variables

The outcome variable considered in this study was disability status of the respondents. Disability was defined, consistent with the NSPD and the Washington Group on Disability Statistics framework, as a long-term physical, sensory, intellectual, or mental impairment which, in interaction with environmental and social barriers, limits an individual’s full and effective participation in society on an equal basis with others. During the survey, respondents were asked whether they experienced any type of disability and were provided with a predefined list of disability categories. Where the reported condition was not included in the predefined list, respondents were allowed to select an “other” category and specify the condition accordingly. The disability categories included autism, physical disability, mental illness, visual impairment, speech impairment, intellectual disability, hearing impairment, combined hearing-visual impairment, cerebral palsy, Down syndrome, multidimensional disability, and other conditions. These categories were developed based on the national framework outlined in The Rights and Protection of Persons with Disabilities Act, 2013 and the Washington Group disability classification framework [19,33].

For analysis, responses were recoded into a binary outcome variable, classifying respondents as persons with disability (coded as 1) if at least one disability was reported, and persons without disability (coded as 0) if no disability was reported. Disability status was determined based on self-report or proxy-report provided by a household member, rather than clinical assessment or medical record verification, consistent with the standard NSPD and Washington Group data collection approach. For respondents unable to self-report, including young children and individuals with severe intellectual disability, autism, or mental illness, responses were provided by a knowledgeable household member, most commonly a parent or primary caregiver. The potential limitations associated with this reporting approach are discussed in the limitations section.

### Explanatory variables

We followed a comprehensive four-stage selection process to select explanatory variables. At the first stage, we searched five databases (Medline, Scopus, CINHAL, Web of Science, Science Direct) along with search with Google and Google Scholar for relevant papers. The factors considered in the papers that were found relevant were then summarized and a list of potential explanatory variables were created at the second stage [30,37,38,42,43]. Subsequently at the third stage, these identified variables were cross-referenced with the survey to verify their availability in the survey data. Those variables that were indeed present were subsequently classified at the fourth stage according to the socio-ecological model of health into three tiers: individual, household, and community-level factors. Individual-level factors included the respondent’s age (0–17 years, 18–59 years, and ≥60 years), years of schooling (treated as continuous variable), gender (male or female), occupation (agriculture, blue-collar work, pink-collar work, white-collar work, student, housewife, and others), marital status (married, unmarried, or widowed/divorced/separated) and religion (Muslim, others). Additionally, at the household level, a variable representing wealth quintiles (poorest, poorer, middle, richer, richest) was included. This variable was formulated by the survey authority via principal component analysis of household asset-related variables within the respondent’s residing household, such as roofing type and ownership of a refrigerator. Moving to community-level factors, we integrated the respondents’ place of residence (urban and rural) and their division (Barishal, Chattogram, Dhaka, Khulna, Mymensingh, Rajshahi, Rangpur, and Sylhet). Two other community-level variables considered were community-level literacy and community-level poverty. The data for these variables were not directly available in the survey; therefore, we constructed them using relevant indicators, specifically respondents’ education status and their household wealth quintile at the PSU level. The calculation procedure involved estimating the mean values of these characteristics within each PSU, classifying individuals with no formal education or only primary education, as well as those belonging to the poorest and poorer wealth quintiles, as the disadvantaged group—consistent with previous literature. Based on these computed values, community-level literacy was categorized as low (≤0.54), moderate (0.54–0.62), and high (>0.62). Community-level poverty was categorized as high (>0.41), moderate (0.16–0.41), low (≤0.15 but >0), and middle or richest communities (0). Detailed procedures for constructing these variables have been published elsewhere [44].

### Statistical analysis

Descriptive statistics were used to provide an overview of both the respondents’ characteristics and the prevalence of disabilities. Prevalence estimates and their corresponding 95% CIs were generated using survey-design-based estimation (the “svy” suite of commands in STATA), accounting for the sampling weights, stratification, and clustering inherent in the two-stage NSPD sampling design. To examine the variation in disability status across the considered explanatory variables and assess their statistical significance, cross-tabulations and chi-square test were performed, respectively. To explore the association of the outcome variable with explanatory variables, multilevel mixed-effect logistic regression model was used. The reasons for selecting the multilevel modeling approach was nested structure of the NSPD data whereas individuals were nested within a household, and households were nested within a PSUs. Earlier research found that multilevel modelling can consider such clustering of data and produced results than simple logistic regression model [45]. In the process of model development, the total sample was stratified into two categories: ages 0–17 and ages 18 and above. These categorizations were based on internationally accepted age guidelines distinguishing children (below 18 years) from adults (18 years and above), consistent with the United Nations Convention on the Rights of the Child and the definition of “child” used in Bangladesh’s own legal and survey frameworks, including the NSPD. This binary split, rather than finer bands (e.g., 18–59 years versus 60 years and above, or 5-/10-year intervals across the full 18–95 range), was adopted for three main reasons. First, the principal analytic distinction of substantive interest for this study was between disability determinants operating in childhood, which are dominated by congenital, developmental, and early-life factors, and those operating across adulthood, where socioeconomic, occupational, and ageing-related pathways predominate; a two-group model was sufficient to capture this contrast while keeping the number of parallel models, and the resulting multiple comparisons, manageable. Second, because disability prevalence in this sample was low (2.79%), further disaggregation of the 18–95 years group (for example, isolating the 60–90/95 years old-age band) substantially reduced the number of disability events within some strata and household/PSU clusters, resulting in unstable multilevel model estimates with implausibly wide confidence intervals for several covariates, particularly at the community level; this is discussed further in the Strengths and limitations section. Third, to still capture age-related variation within the broad 18–95 years adult group, respondents’ age in years was retained and modelled as a continuous explanatory variable within the adult-group models, so that the gradient of risk from young adulthood through to advanced old age (including the 65–90/95 years old-age band raised above) is reflected in the reported age coefficient rather than collapsed into a single undifferentiated category. For each of these age groups, a progressive model building technique was used, involving the creation of four distinct models: null model, individual-level model, household-level model, and full model. The null model included only the status of disabilities, whereas in the individuals level model, respondents’ characteristics were considered. Households level characteristics were incorporated at the household level model along with individual level factors. The final model included all individual, households and community level factors. Where appropriate, the presence of multicollinearity was checked among variables using variance inflation factors (VIF) at a cut-off point of 5. If a VIF was 5 or more, the relevant variable was deleted and the model was run again. The results are reported as Odds Ratios, accompanied by their corresponding 95% Confidence Intervals (95% CI). The statistical analyses were conducted using STATA/SE 14.0 (Stata Corp LP, College Station, Texas, United States of America).

Ethics statement: The NSPD survey we analyzed was reviewed and approved by the Ethics Committee of the Bangladesh Bureau of Statistics. Informed written consent was obtained from each respondent (or their legal guardian for respondents aged <18 years) before collecting data. We obtained de-identified data from the BBS by submitting a research proposal. Therefore, there is no need for any additional ethical approval.

## Results

### Background characteristics of the respondents

The background characteristics of the respondents are presented in Table 1. Out of the 155,025 individuals whose data were analyzed, 4,293 individuals had one or more form of disabilities, constituting approximately 2.79% (95% CI, 2.69-2.91) prevalence of disabilities. Disability prevalence was higher among males (3.27%) than females (2.31%) and rose markedly with age, from 1.67% among children aged 0–17 years, to 2.55% among adults aged 18–59 years, to 8.49% among adults aged 60 years and above (S1 Table). More than half of the respondents were aged 18–59 years (56%), while one-thirds (35%) of the total respondents aged 17 years or less. Roughly 26% of the total respondents classified as students, while housewives made up a further sizeable share of the sample, together constituting the largest occupation categories among respondents. A significant majority, accounting for over 78% of the total respondents, reported residing in rural areas, while around 24% indicated Dhaka as their division of residence, and respondents were distributed across the five household wealth quintiles in line with the quintile-based construction of this variable.

**Table 1:** Background characteristics of the respondents, N = 155,025, Bangladesh, 2021.

Characteristics	Percentage	(95% CI)
Respondents’ gender		
Male	49.95	49.73–50.16
Female	50.05	49.84–50.27
Respondents’ age (in years)		
Mean age (±SD)	28.89 (±19.97)	
0–17	34.78	34.44–35.14
18–59	55.93	55.61–56.25
≥60	9.29	9.09–9.49
Respondents’ year of schooling mean (SD)	7.37(±12.05)	
Respondents’ occupation		
Agriculture	8.14	7.80–8.50
Blue collar worker ^a^	9.71	9.28–10.15
Pink collar worker ^b^	5.31	5.11–5.52
White collar worker ^c^	8.95	8.64–9.26
Student	26.70	26.41–26.99
Housewives	25.23	24.97–25.49
Others^*^	15.96	15.72–16.21
Respondents’ marital status		
Married	51.47	51.15–51.79
Unmarried	43.07	42.75–43.39
Widow/Divorce/Separate	5.46	5.32–5.61
Respondents’ religion		
Muslim	90.26	88.62–91.69
Others^**^	9.74	8.31–11.38
Wealth quintile		
Poorest	20.00	18.68–21.39
Second	20.00	19.29–20.73
Middle	20.00	19.29–20.73
Fourth	20.00	19.07–20.96
Richest	20.00	18.78–21.27
Place of residence		
Rural	78.08	77.68–78.48
Urban	21.92	21.52–22.32
Division of residence		
Barishal	6.10	5.96–6.25
Chattogram	18.81	18.42–19.22
Dhaka	23.97	23.44–24.50
Khulna	10.76	10.55–10.97
Mymensingh	8.26	8.05–8.49
Rajshahi	13.06	12.78–13.34
Rangpur	11.66	11.40–11.92
Sylhet	7.38	6.30–8.63
Community level literacy		
Low	25.94	23.10–29.00
Moderate	25.37	22.42–28.56
High	48.69	45.29–52.09
Community level poverty		
High poverty	49.22	46.14–52.31
Moderate poverty	27.74	24.84–30.85
Low poverty	10.10	8.20–12.37
Middle or richest community	12.94	11.39–14.65

Note: ^a^Blue collar worker means [factory/manufacturing workers/labour, transportation / communication workers, day labor (non-agriculture), auto / cng / tempo driver, rickshaw driver / van driver / boatman, poultry /animal husbandry for business, fishery or aquaculture and fisherman].

b Pink collar worker means [small business (capital up to taka 1000), business (capital over taka 10000), kabiraj/ojha/spiritual physician, village doctor and homeopathy doctor].

c White collar worker means [teacher, lawyer /journalist /doctor/engineer, government employee/officer, private/Ngo employee / officer, handicraft/cottage industry, weaver/blacksmith/potter/goldsmith/service, imam/ priest, family helper and housemaid/servant].

* family helper, servant, looking for work, unable for work, beggar, no work and not looking for work, and other unnamed occupations; **Hindu, Buddhism, Christianity, etc.

### Prevalence of different type of disabilities in Bangladesh

Table 2 presents the distribution of persons with disabilities according to the type of disability. Among the 4,293 persons with disabilities, the most prevalent form was physical impairment (42.44%, 95% CI, 40.79-44.11), followed by visual impairment (14.11%, 95% CI, 13.03-15.25) and multidimensional disability, i.e., more than one concurrent type of disability (11.60%, 95% CI, 10.55-12.75). Mental illness (8.38%, 95% CI, 7.54-9.30), hearing impairment (6.97%, 95% CI, 6.15-7.90), and intellectual disability (4.92%, 95% CI, 4.25-5.69) were also reported by a sizeable proportion of persons with disabilities, whereas autism spectrum disorder (1.60%), Down syndrome (1.22%), and combined hearing-visual impairment (0.46%) were comparatively rare.

**Table 2:** Prevalence of different type of disabilities in Bangladesh, N = 4,293.

Type of disabilities	Prevalence, 95% CI
Autism or autism spectrum disorder	1.60 (1.25–2.05)
Physical impairment	42.44 (40.79–44.11)
Mental illness	8.38 (7.54–9.30)
Visual impairment	14.11 (13.03–15.25)
Speech impairment	4.08 (3.48–4.77)
Intellectual disability	4.92 (4.25–5.69)
Hearing impairment	6.97 (6.15–7.90)
Hearing-visual impairment	0.46 (0.30–0.71)
Cerebral palsy	2.77 (2.31–3.32)
Down syndrome	1.22 (0.91–1.62)
Multidimensional disability	11.60 (10.55–12.75)
Others	1.45 (1.06–1.96)

### Prevalence of disability across individual, household and community level factors

The presentation of disability prevalence across the considered explanatory variables are presented in the S1 Table. A higher prevalence of disabilities was found among males, individuals aged 60 or older, and those classified as widowed, divorced, or separated. Higher prevalence of disabilities was found among individuals in the Khulna and Rangpur divisions. Significant variations in the occurrence of disabilities were found across individual, household, and community-level factors considered as explanatory variables except for respondents’ religion and community level literacy. Our further exploration on the prevalence of disabilities at the district level (second administrative level in Bangladesh) presents evidence of district level variations in prevalence of disabilities (Fig 1).

**Fig 1:**
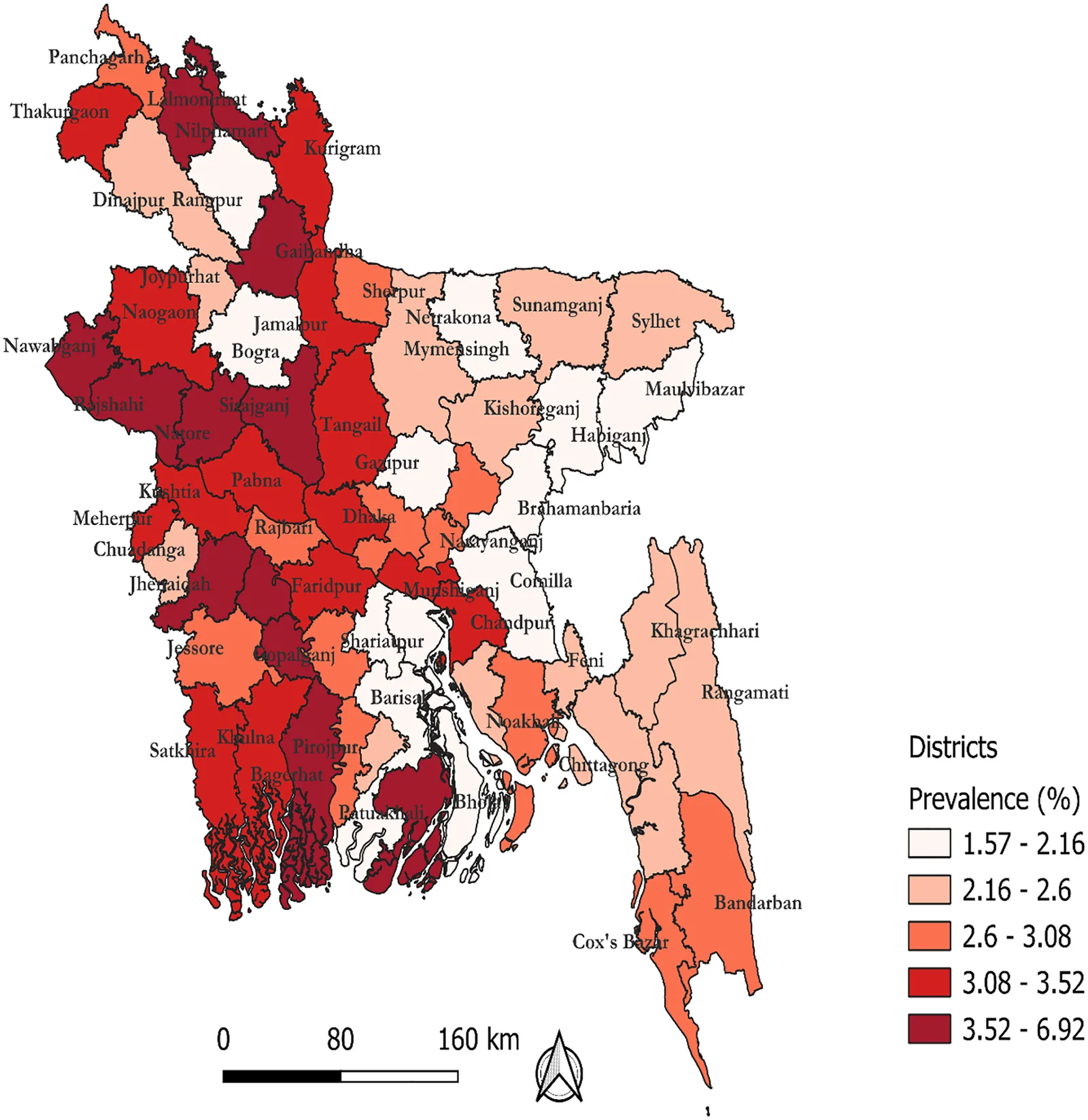
District-wise prevalence of disability in Bangladesh. The map was generated by the authors using ArcGIS version 10. The base-map shapefile was sourced from The Humanitarian Data Exchange (https://data.humdata.org/dataset/cod-ab-bgd) and is licensed under the Creative Commons Attribution for Intergovernmental Organisations (CC BY-IGO) license. The data have been adapted by the authors, and the licensor does not endorse this work.

### Model selection

The factors associated with disabilities were evaluated using a multilevel mixed-effect logistic regression model. Models were constructed for two distinct age groups: 0–17 years (first model) and 18 years or older (second model). For each sample category, a total of four models were run, with the best model determined through a comparison of the Intra Class Correlation (ICC), Akaike Information Criterion (AIC) and Bayesian Information Criterion (BIC). The best model was one which had the lowest ICC, AIC and BIC. The full model met these criteria, as such it was selected as the best fitted model (Table 3).

**Table 3:** Random intercept model of variations at cluster/community level for persons with disabilities in Bangladeshi population, NSPD 2021.

Random effects (measure of variation for disability)^a^	Individuals aged 0–17 years ^±^				Individual aged 18–95 years ^±^			
	Null model	Individual level model	Individual and household level model	Individual, household, and community level model	Null model	Individual level model	Individual and household level model	Individual, household, and community level model
Intra-class correlation (ICC, %)	7%	6.5%	4%	3.4%	7%	6.5%	5%	4.5%
Model summary								
Akaike’s Information Criterion (AIC)	9182.58	7599.98	7589.71	7574.54	29762.94	24765.87	24556.75	24461.72
Bayesian Information Criteria (BIC)	9200.37	7642.90	7614.13	7580.57	29781.98	24870.60	24718.62	24707.36

Notes: ^±^ Models are based on multilevel mixed-effects logistic regression.

### Individuals, households, and community level characteristics associated with disabilities

The results of final models for both groups are presented in Table 4 whereas the results of full models are presented in the S2 and S3 Tables. The likelihood of disabilities was 1.31 times higher (aOR: 1.31, 95% CI: 1.13–1.52) among respondents with female aged 0–17 years as compared to respondents with male. The likelihoods of disabilities were found to be increased by 1% and 12% point for each year increase of education for the respondents aged 0–17 years and 18 years or more, respectively. Similarly, in occupation categories, the likelihood of disabilities were higher among blue-collar workers (aOR: 1.43, 95% CI: 1.22–1.67), students (aOR: 1.75, 95% CI: 1.35–2.27), and housewives (aOR: 1.77, 95% CI: 1.49–2.10) compared to individuals engaged in agriculture. Conversely, lower likelihoods of disabilities were found among white-collar workers (aOR: 0.79, 95% CI: 0.67–0.93) and individuals falling into the “others” category (aOR: 0.12, 95% CI: 0.10–0.13). The likelihood of disabilities was 70% lower (aOR: 0.30, 95% CI: 0.12–0.73) among unmarried individuals aged 0–17 years and 59% lower (aOR: 0.41, 95% CI: 0.36–0.47) among the unmarried individuals aged 18 and more as compared to their married counterparts. Moreover, the likelihood of disabilities was found to be increased with progressing household wealth quintiles, indicating a gradient relationship. Individuals residing in the Rangpur, Rajshahi, and Khulna divisions reported lower likelihoods of disabilities compared to those residing in the Barishal division. Notably, residing in a community with moderate (aOR: 1.22, 95% CI: 1.06–1.41) or high (aOR: 1.38, 95% CI: 1.20–1.58) level of literacy was found associated with a 122–138% higher likelihood of disabilities as compared to the respondents resided in the community lower literacy levels.

**Table 4:** Individual, households and community level factors associated with disability in Bangladesh.

Characteristics	Odds ratio (95% Confidence Interval)	
	Individuals aged 0–17 years	Individual aged 18–95 years
**Respondents’ age (in years)**	0.98 (0.96–1.00)	0.99 (0.99–1.00)
**Respondents’ gender**		
Male	1.00	1.00
Female	1.31 (1.13–1.52) ^***^	1.06 (0.95–1.19)
**Respondents’ year of schooling**	1.01(1.00–1.02) ^***^	1.12(1.11–1.13) ^***^
**Respondents’ occupation**		
Agriculture	na	1.00
Blue collar worker	na	1.43 (1.22–1.67) ^***^
Pink collar worker	na	0.99 (0.82–1.20)
White collar worker	na	0.79 (0.67–0.93) ^***^
Student	na	1.75(1.35–2.27) ^***^
Housewives	na	1.77 (1.49–2.10) ^***^
Others	na	0.12 (0.10–0.13) ^***^
**Respondents’ marital status**		
Married	1.00	1.00
Unmarried	0.30 (0.12–0.73) ^***^	0.41 (0.36–0.47) ^***^
Widow/Divorce/Separate	0.18 (0.02–1.62)	0.94 (0.83–1.06)
**Wealth Quintile**		
Poorest	1.00	1.00
Second	1.22(0.99–1.50)	1.20 (1.08–1.33) ^***^
Middle	1.38 (1.10–1.73) ^**^	1.32 (1.17–1.48) ^***^
Fourth	1.56 (1.22–2.01) ^***^	1.52 (1.33–1.73) ^***^
Richest	1.92 (1.40–2.62) ^***^	1.68 (1.43–1.98) ^***^
**Place of residence**		
Rural	1.00	1.00
Urban	1.06 (0.81–1.40)	0.96 (0.81–1.13)
**Division of residence**		
Barishal	1.00	1.00
Chattogram	1.05 (0.75–1.49)	0.93 (0.75–1.14)
Dhaka	0.77 (0.55–1.10)	0.84 (0.68–1.04)
Khulna	0.64 (0.44–0.93) ^**^	0.64 (0.52–0.80) ^***^
Mymensingh	0.84 (0.58–1.22)	0.94 (0.75–1.18)
Rajshahi	0.69 (0.49–0.98) ^**^	0.62 (0.51–0.76) ^***^
Rangpur	0.65 (0.46–0.91) ^**^	0.58 (0.48–0.72) ^***^
Sylhet	1.06 (0.73–1.54)	1.24 (0.98–1.56)
**Community level literacy**		
Low	1.00	1.00
Moderate	0.77 (0.61–0.97) ^**^	1.22 (1.06–1.41) ^**^
High	0.92 (0.73–1.16)	1.38 (1.20–1.58) ^***^
**Community level poverty**		
High poverty	1.00	1.00
Moderate poverty	0.88 (0.71–1.09)	1.06 (0.93–1.21)
Low poverty	0.76 (0.54–1.08)	1.00 (0.82–1.23)
Middle or richest community	0.56 (0.36–0.86) ^***^	1.13 (0.87–1.48)

Note: ^**^ p < 0.05, ^***^ p < 0.01, na = Not applicable.

## Discussion

The aims of this study were twofold: to estimate the national-level burden of disabilities in Bangladesh and to identify its individual, household, and community-level correlates, separately for children (0–17 years) and adults (18–95 years). Findings indicate that approximately 3% of Bangladesh’s total population experiences one or more form of disabilities. The most prevalent forms were physical disability, followed by visual impairment and multidimensional disability. In the unadjusted descriptive analysis, disability was more prevalent among males, among older respondents (rising from 1.67% among those aged 0–17 years to 8.49% among those aged 60 years and above), among widowed, divorced, or separated individuals, and among residents of the Khulna, Rajshahi, and Rangpur divisions. However, after adjusting for individual, household, and community-level factors in the multilevel regression models, several of these patterns changed direction or lost significance: adjusted odds of disability were lower, not higher, among unmarried individuals and among residents of Khulna, Rajshahi, and Rangpur, indicating that part of the unadjusted excess in these groups was explained by their other socio-demographic characteristics rather than these factors independently. In contrast, higher adjusted odds of disability persisted among girls aged 0–17 years (relative to boys), and, in the adult (18–95 years) model, among individuals with more years of schooling, higher household wealth quintiles, housewives, students, blue-collar workers, and residents of communities with moderate-to-high literacy. These findings, drawn from an advanced multilevel statistical model applied to a large, nationally representative sample that accounted for a comprehensive range of individual, household, and community-level factors, are considered robust and are used below to draw out their implications for national policy and clinical/public health practice.

The reported prevalence of disabilities in this study was approximately 3% which is significantly lower than the global prevalence of disabilities 16% reported by the WHO in 2022 [1]. Notably, prevalence of disabilities exhibited substantial variation at the national level, ranging from 1.0% to 18.0% [5,39]. This discrepancy highlights a remarkable advancement within the country’s context, although the absolute number of individuals struggling with disabilities remains significant, encompassing an estimated 4.8 million people. The underlying factors contributing to this relatively lower prevalence of disabilities in Bangladesh can be attributed to the comparatively smaller proportion of the aging population and a higher concentration of rural residents [46,47]. It is essential, however, to acknowledge the ongoing rapid shifts observed in both these indicators within the nation. The surge in urban dwellers and the aging population signals the potential for an escalation in the prevalence of disabilities in the coming years [47]. This projection can be attributed to a surge in accidents stemming from urbanization and industrialization, alongside the migration from rural to urban settings [7,48]. Additionally, heightened survival rates subsequent to accidents leading to physical impairments, coupled with increased life expectancy amidst various health conditions, collectively contribute to this trajectory [12,49,50].

These characteristics are also exhibiting a more pronounced presence among individuals with comparatively higher educational backgrounds and white-collar occupations in Bangladesh and other LMICs [30,51]. Moreover, the gradual increase of likelihood of disabilities with the increase of household wealth quintiles provide further justification for the aforementioned possibilities. These observation can be justified by the fact that people with increasing education and wealth quintile, which are even interrelated and comes together, are more likely to report disabilities because of their increased awareness and accessibility to healthcare services as compared to their counterpart with poor education and resided in household with comparatively disadvantaged wealth quintile [52]. Moreover, heightened educational levels and enhanced socioeconomic status often correlate with increased engagement in activities involving greater risk, whether due to occupation-related hazards or participation in recreational pursuits [51]. Importantly, although both males and females participate in high-risk occupations, males typically represent the majority, which contributes to their higher likelihood of disabilities, as reported in this study and in previous studies conducted in LMICs [47,53,54]. Paradoxically, despite having improved access to healthcare services, there is a prevailing norm that prioritize the receipt of health care services based on immediate needs, seeking medical attention solely when unwell, rather than prioritizing services that hold potential for sustained long-term health benefits [52,55]. It allows certain conditions to evolve into disabilities over time. Moreover, the extended lifespan typically associated with enhanced educational and economic circumstances can influence individuals to age-associated disabilities [52]. Psychosocial stressors, a facet frequently encountered within higher socioeconomic tiers, might also contribute to mental health challenges that can, over time, manifest as disabilities [56].

The existing burden of non-communicable diseases (NCDs) in Bangladesh, as like other LMICs, is particularly pronounced among individuals with higher educational achievements, residing in urban areas, and positioned within the upper levels of wealth quintiles [47,57,58]. The primary underlying reasons are an increased dependency on western food and a higher rate of overweight/obesity [59]. Individuals belonging to these categories tend to adopt more hygienic lifestyles, often allocating less time to tending to their physical well-being. People with these characteristics are more likely to report disabilities, as indicated in this study and in other studies conducted in LMICs [60–63]. A recent Bangladeshi study using the same cohort of disabled individuals we analyzed also reported that around half of them have comorbidities, with highly prevalent comorbidity being chronic conditions, including NCDs [64]. The pathways of a higher rate of disabilities following chronic conditions could be linked with a higher rate of undiagnosed and untreated chronic conditions, with treatment received when disabilities has already onset.

However, lower likelihood of disabilities among individuals resided in the Khulna, Rajshahi and Rangpur divisions as well as lower prevalence of disabilities in certain district of Bangladesh indicate different perspective of these observations. The aforementioned divisions as well as districts with low prevalence of disabilities are characterized by lower prevalence of urbanization and industrialization, with resident there are primarily engaged with agriculture [7,65]. This pattern is consistent with the broader development profile of these divisions within Bangladesh: Khulna, Rajshahi and Rangpur have historically reported comparatively lower per-capita income, lower rates of formal-sector employment, and more limited concentration of tertiary-level health facilities than divisions such as Dhaka and Chattogram, meaning many health conditions that would otherwise be diagnosed and recorded as disabling may instead go unrecognised. The same underlying mechanism, namely lower contact with the formal health system, plausibly also explains the comparatively lower reported disability prevalence among the unmarried, among rural residents, and among the poorest wealth quintile observed in this study, since each of these groups has relatively constrained access to diagnostic healthcare in the Bangladeshi context. These findings should therefore be interpreted as reflecting under-ascertainment of disability in lower-resource settings rather than necessarily a true lower biological risk, an interpretation directly relevant for national policy: disability-screening and outreach services may need to be prioritised in these comparatively underserved divisions to ensure equitable identification and support of persons with disabilities across the country. They are also less likely to access healthcare services indicating risk of any health conditions are primary undiagnosed and untreated indicating increased likelihoods of disabilities [52].

We found a very higher likelihood of disabilities among widowed, divorced, or separated as compared to the normal married or unmarried individuals. This could be attributed to factors such as economic hardship, social isolation, depression, anxiety, and chronic health conditions [15,66]. Unmarried women may face financial challenges, leading to stress and health issues [67]. They may also have limited social support, contributing to stress-related health problems. The loss of a spouse or the end of a marriage can trigger depression and anxiety, potentially leading to disabilities [68]. Unmarried women might also have a higher prevalence of chronic health conditions like heart disease, stroke, and diabetes, which can further increase the risk of disabilities [16,69]. However, there could be other factors involved and further research is necessary to fully understand this phenomenon.

The policy implications of this study is that national actual burden of persons with disabilities is higher with nearly 3% disability which is belong to around 4.8 million population [41]. However, the higher likelihoods of disabilities are higher among individuals with higher education, higher wealth quintile, housewives, students and blue-collar workers, among them awareness about health is usually higher, indicate possibility of underreporting disabilities among individuals of comparatively disadvantaged position in response to these characteristics [70]. These suggest need for awareness building programs to ensure disabilities are reported. Healthcare service should be strengthened to ensure access of healthcare services when needed. Policies and programs are also necessary aimed at tackling the multifaceted challenges encountered by persons with disabilities.

### Clinical and public health practice implications

Beyond broad policy recommendations, these findings translate into several concrete implications for clinical and public health practice, which differ for the two age groups examined. For children (0–17 years), the higher adjusted odds of disability among girls warrants routine, sex-disaggregated developmental and disability screening within existing maternal, neonatal, and child health (MNCH) and Expanded Programme on Immunization (EPI) contact points, so that early impairments among girls are not under-identified relative to boys; frontline health workers and school health programmes should be equipped with simple, validated screening tools (e.g., the Washington Group/UNICEF Child Functioning Module) to support early referral to rehabilitation and special-education services. For adults (18–95 years), the sharp rise in prevalence from 2.55% among those aged 18–59 years to 8.49% among those aged 60 years and above supports the integration of disability and functional-status screening into geriatric and NCD care pathways at the primary healthcare level, given that stroke, other NCDs, and age-related sensory and physical decline are established contributors to late-life disability in this setting. The elevated odds observed among blue-collar workers point to a need for strengthened occupational health and safety regulation and injury-prevention programmes in manufacturing, transport, and construction sectors, together with workplace-based rehabilitation and return-to-work support for workers who acquire a disability. The concentration of higher adjusted odds among more educated, wealthier, and urban-adjacent population groups, alongside comparatively lower reported prevalence in Khulna, Rajshahi, and Rangpur divisions and among the poorest wealth quintile, is consistent with under-ascertainment of disability where diagnostic health-service contact is limited; clinically, this argues for proactively extending community-based disability identification, rather than relying on passive facility-based diagnosis, into rural and lower-resource divisions through community clinics and Union Health and Family Welfare Centres, so that disability case-finding is not inadvertently biased toward populations that are already better connected to the health system. Embedding these screening and referral functions within existing primary healthcare and social-service infrastructure, rather than creating parallel systems, offers a feasible pathway to translate the correlates identified in this study into earlier detection, timely rehabilitation, and more equitable access to disability-support services across Bangladesh.

### Strengths and limitations

This study has several strengths. As far we know this is the first study in Bangladesh that explored the national level burden of disabilities and its corelates by analyzing large nationally representative households survey. Recognized procedure was applied to measure disabilities. Data were analyzed by comprehensive statistical modelling with hierarchical structure of the data and sampling weights were considered in all analysis. Therefore, they findings are robust, and can be used in developing national level policies and programs.

However, the primary limitations of this study were the analysis of cross-sectional data, which limited our capacity to established causality and the findings were correlational only. Data were collected by asking questions to the respondents with no chance of validation. This indicates possibility of presence recall bias, though any of such bias is likely to be random. Relatedly, disability status was based on self- or proxy-report rather than confirmation against medical records or clinical diagnosis, and for some conditions, such as mental illness, intellectual disability, and autism, a household member sometimes responded on behalf of the affected individual; this may have introduced some misclassification, although the standardised list-based question format used by the NSPD is designed to minimise such bias and is widely used internationally for disability enumeration. The survey design also did not allow us to determine whether reported disabilities were congenital, acquired through degeneration or chronic disease, or related to comorbid non-communicable conditions, nor whether respondents were currently receiving treatment or rehabilitation, or how long they had been living with the disability; these are important questions that future studies, ideally incorporating clinical verification or linkage to health records, should address. The NSPD also did not capture severity gradation (e.g., mild, moderate, severe) for most disability types, nor sufficient detail on the specific underlying NCD or comorbidity associated with each disability type to allow direct disability-type-by-disability-type comparison with other LMICs (for example, to explain why hearing impairment was less prominent in our sample than in some other LMIC settings); these remain important targets for future, more detailed surveillance. Moreover, other than the factors adjusted in the model, health and environmental factors can contribute to the onset in disabilities as such they are important to be adjusted in the model. However, these data were not available in the survey, limiting us to do so. Although stratifying the analysis by ages below 18 and 18 years or older provides an opportunity to identify correlates separately for children and adults, it would have been beneficial to further narrow these categories into smaller intervals-such as 5- or 10-year age groups. However, this was not feasible, as such disaggregation would have resulted in very small sample sizes within each category, preventing the development of models capable of producing reliable estimates. Similarly, analyzing data by specific types of disability rather than grouping all disability categories together as a binary outcome would have yielded more nuanced insights. Yet this approach was also not possible due to the limited number of observations for each disability type. However, regardless of these limitations the findings of this study will contribute to the national level policies and program development.

## Conclusions

This study reported that approximately three out of every hundred people in Bangladesh are now facing disabilities, with physical disability and visual impairment being the most common types. Additionally, about one in ten individuals with disabilities reported experiencing multiple forms of impairment. A higher likelihood of disabilities was found among individuals with increasing years of education, higher wealth quintiles, housewives, students, and those in blue-collar occupations. People residing in the Khulna, Rajshahi, and Rangpur divisions had a lower likelihood of disabilities compared to those in Barishal, indicating regional variations in disability prevalence across administrative divisions, with lower reported prevalence in these divisions likely reflecting under-ascertainment linked to comparatively limited healthcare access rather than genuinely lower risk. Accordingly, we recommend that the Ministry of Health and Family Welfare and the Department of Social Services, in collaboration with local government institutions, prioritise community-based disability screening and referral services in the Khulna, Rajshahi, and Rangpur divisions and among rural, unmarried, and lower wealth-quintile populations nationally, using existing community clinics and Union Health and Family Welfare Centres as delivery points, so that under-identified persons with disabilities can be enrolled in existing government disability allowance, rehabilitation, and assistive-device programmes administered by the Department of Social Services. Furthermore, prospective cohort studies should be conducted, considering a broad range of factors, to gain a more nuanced understanding of disabilities and their associated factors in Bangladesh.

## Supporting information

S1 Table Distribution of disability and individual, household and community level factors, Bangladesh. (DOCX)

S2 Table Socio-demographic characteristics associated with disability among children those aged 0–17 in Bangladesh. (DOCX)

S3 Table Socio-demographic characteristics associated with disability among individuals aged 18–95 years in Bangladesh. (DOCX)

## Abbreviations

PWD: Persons with disabilities
WHO: World Health Organization
LMICs: Low and middle-income countries
VIF: Variance Inflation Factors
BBS: Bangladesh Bureau of Statistics
aOR: Adjusted Odds Ratios
AIC: Akaike Information Criteria
BIC: Bayesian Information Criteria
