# Supplementary material for: National burden of disabilities in Bangladesh and its socio-demographic correlates: A 2021 cross-sectional analysis
Source: PLOS Glob Public Health. 2026 Oct 8;6(10):e0007463. doi: 10.1371/journal.pgph.0007463 (PMC13648970; doi:10.1371/journal.pgph.0007463)
Supplement: Supplementary file 1 [file pgph.0007463.s001.docx]

S1 Table: Distribution of disability and individual, household and community level factors, Bangladesh.

| Characteristics | Persons with disability | | p-value^+^ |
| --- | --- | --- | --- |
|  | Yes | No |  |
| Respondents’ gender |  |  |  |
| Male | 3.27 | 96.73 | p<0.001 |
| Female | 2.31 | 97.69 |  |
| Respondents’ age (in years) |  |  |  |
| 0-17 | 1.67 | 98.33 | p<0.001 |
| 18-59 | 2.55 | 97.45 |  |
| ≥60 | 8.49 | 91.51 |  |
| Respondents’ year of schooling | 3.89 | 7.48 | p<0.001 |
| Respondents’ occupation |  |  |  |
| Agriculture | 3.29 | 96.71 | p<0.001 |
| Blue collar worker | 1.95 | 98.05 |  |
| Pink collar worker | 2.01 | 97.99 |  |
| White collar worker | 2.73 | 97.27 |  |
| Student | 1.18 | 98.82 |  |
| Housewives | 1.30 | 98.70 |  |
| Others | 8.41 | 91.59 |  |
| Respondents’ marital status |  |  |  |
| Married | 2.61 | 97.39 | p<0.001 |
| Unmarried | 2.27 | 97.73 |  |
| Widow/Divorce/Separate | 8.66 | 91.34 |  |
| Respondents’ religion |  |  |  |
| Muslim | 2.77 | 97.23 | p=0.139 |
| Others | 3.01 | 96.99 |  |
| Wealth quintile |  |  |  |
| Poorest | 3.79 | 96.21 | p<0.001 |
| Second | 3.07 | 96.93 |  |
| Middle | 2.77 | 97.23 |  |
| Fourth | 2.37 | 97.63 |  |
| Richest | 1.98 | 98.02 |  |
| Place of residence |  |  |  |
| Rural | 2.89 | 97.11 | p<0.001 |
| Urban | 2.44 | 97.56 |  |
| Division of residence |  |  |  |
| Barishal | 2.42 | 97.58 | p<0.001 |
| Chattogram | 2.41 | 97.59 |  |
| Dhaka | 2.51 | 97.49 |  |
| Khulna | 3.61 | 96.39 |  |
| Mymensingh | 2.45 | 97.55 |  |
| Rajshahi | 3.30 | 96.7 |  |
| Rangpur | 3.54 | 96.46 |  |
| Sylhet | 2.15 | 97.85 |  |
| Community level literacy |  |  |  |
| Low | 2.73 | 97.27 | p=0.3002 |
| Moderate | 2.95 | 97.05 |  |
| High | 2.75 | 97.25 |  |
| Community level poverty |  |  |  |
| High poverty | 2.97 | 97.03 | p<0.001 |
| Moderate poverty | 2.84 | 97.16 |  |
| Low poverty | 2.65 | 97.35 |  |
| Middle or richest community | 2.15 | 97.85 |  |

Note: All values are presented with row percentage in supplementary table 2, ^+^p-value obtained from the chi square test except variable respondent’s year of schooling where p-value obtained from proportion test.
