# Supplementary material for: National burden of disabilities in Bangladesh and its socio-demographic correlates: A 2021 cross-sectional analysis
Source: PLOS Glob Public Health. 2026 Oct 8;6(10):e0007463. doi: 10.1371/journal.pgph.0007463 (PMC13648970; doi:10.1371/journal.pgph.0007463)
Supplement: Supplementary file 2 [file pgph.0007463.s002.docx]

S2 Table: Socio-demographic characteristics associated with disability among children those aged 0–17 in Bangladesh

| Characteristics | Null model | Individual level model | Household level model | Full model |
| --- | --- | --- | --- | --- |
|  |  | OR (95% CI) | OR (95% CI) | OR (95% CI) |
| Respondents’ age (in years) |  | 0.99 (0.97–1.01) | 0.98 (0.96–1.00) | 0.98 (0.96–1.00) |
| Respondents’ gender |  |  |  |  |
| Male |  | 1.00 | 1.00 | 1.00 |
| Female |  | 1.35 (1.17–1.56) ^***^ | 1.32 (1.14–1.53) ^***^ | 1.31 (1.13–1.52) ^***^ |
| Respondents’ year of schooling |  | 1.02(1.01–1.02) ^***^ | 1.01(1.01–1.02) ^***^ | 1.01(1.00–1.02) ^***^ |
| Respondents’ marital status |  |  |  |  |
| Married |  | na | 1.00 | 1.00 |
| Unmarried |  | na | 0.32 (0.13–0.78) ^**^ | 0.30 (0.12–0.73) ^***^ |
| Widow/Divorce/Separate |  | na | 0.17 (0.02–1.57) | 0.18 (0.02–1.62) |
| Wealth Quintile |  |  |  |  |
| Poorest |  | na | 1.00 | 1.00 |
| Second |  | na | 1.20 (0.97–1.47) | 1.22(0.99–1.50) |
| Middle |  | na | 1.29 (1.04–1.61) ^**^ | 1.38 (1.10–1.73) ^**^ |
| Fourth |  | na | 1.35 (1.07–1.68) ^**^ | 1.56 (1.22–2.01) ^***^ |
| Richest |  | na | 1.48 (1.16–1.88) ^***^ | 1.92 (1.40–2.62) ^***^ |
| Place of residence |  |  |  |  |
| Rural |  | na | na | 1.00 |
| Urban |  | na | na | 1.06 (0.81–1.40) |
| Division of residence |  |  |  |  |
| Barisal |  | na | na | 1.00 |
| Chittagong |  | na | na | 1.05 (0.74–1.49) |
| Dhaka |  | na | na | 0.77 (0.55–1.10) |
| Khulna |  | na | na | 0.64 (0.44–0.93) ^**^ |
| Mymensingh |  | na | na | 0.84 (0.58–1.22) |
| Rajshahi |  | na | na | 0.69 (0.49–0.98) ^**^ |
| Rangpur |  | na | na | 0.64 (0.46–0.91) ^**^ |
| Sylhet |  | na | na | 1.06 (0.73–1.54) |
| Community level literacy |  |  |  |  |
| Low |  | na | na | 1.00 |
| Moderate |  | na | na | 0.77 (0.61–0.97) ^**^ |
| High |  | na | na | 0.92 (0.73–1.16) |
| Community level poverty |  |  |  |  |
| High poverty |  | na | na | 1.00 |
| Moderate poverty |  | na | na | 0.88 (0.71–1.09) |
| Low poverty |  | na | na | 0.76 (0.54–1.08) |
| Middle or richest community |  | na | na | 0.56 (0.36–0.86) ^***^ |
| Model statistics |  |  |  |  |
| ICC | 7% | 6.5% | 4% | 3.4% |
| AIC | 9182.58 | 7599.98 | 7589.71 | 7574.54 |
| BIC | 9200.37 | 7642.90 | 7614.13 | 7580.57 |

Note: ^**^ p<0.05, p<0.01, na= Not applicable
