# Supplementary material for: National burden of disabilities in Bangladesh and its socio-demographic correlates: A 2021 cross-sectional analysis
Source: PLOS Glob Public Health. 2026 Oct 8;6(10):e0007463. doi: 10.1371/journal.pgph.0007463 (PMC13648970; doi:10.1371/journal.pgph.0007463)
Supplement: Supplementary file 3 [file pgph.0007463.s003.docx]

S3 Table: Socio-demographic characteristics associated with disability among individuals aged 18–95 years in Bangladesh (N= 100,853)

| Characteristics | Null model | Individual level model | Household level model | Full model |
| --- | --- | --- | --- | --- |
|  |  | OR (95% CI) | OR (95% CI) | OR (95% CI) |
| Respondents’ age (in years) |  | 1.01 (1.00–1.01) ^***^ | 1.00 (1.00–1.00) ^**^ | 0.99 (0.99–1.00) |
| Respondents’ gender |  |  |  |  |
| Male |  | 1.00 | 1.00 | 1.00 |
| Female |  | 1.06 (0.96–1.16) | 1.05 (0.94–1.18) | 1.06 (0.95–1.19) |
| Respondents’ year of schooling |  | 1.13(1.12–1.14) ^***^ | 1.11(1.10–1.13) ^***^ | 1.12(1.11–1.13) ^***^ |
| Respondents’ occupation |  |  |  |  |
| Agriculture |  | 1.00 | 1.00 | 1.00 |
| Blue collar worker |  | 1.46 (1.24–1.71) ^***^ | 1.47 (1.25–1.73) ^***^ | 1.43 (1.22–1.67) ^***^ |
| Pink collar worker |  | 1.11 (0.92–1.34) | 1.01 (0.84–1.23) | 0.99 (0.82–1.20) |
| White collar worker |  | 0.78 (0.67–0.92) ^***^ | 0.82 (0.70–0.96) ^**^ | 0.79 (0.67–0.93) ^***^ |
| Student |  | 1.18 (0.91–1.51) | 1.75 (1.35–2.27) ^***^ | 1.75(1.35–2.27) ^***^ |
| Housewives |  | 2.07 (1.75–2.45) ^***^ | 1.81 (1.52–2.15) ^***^ | 1.77 (1.49–2.10) ^***^ |
| Others |  | 0.10 (0.09–0.12) ^***^ | 0.12 (0.11–0.14) ^***^ | 0.12 (0.10–0.13) ^***^ |
| Respondents’ marital status |  |  |  |  |
| Married |  | na | 1.00 | 1.00 |
| Unmarried |  | na | 0.42 (0.37–0.48) ^***^ | 0.41 (0.36–0.47) ^***^ |
| Widow/Divorce/Separate |  | na | 0.93 (0.83–1.06) | 0.94 (0.83–1.06) |
| Wealth Quintile |  |  |  |  |
| Poorest |  | na | 1.00 | 1.00 |
| Second |  | na | 1.19 (1.07–1.32) ^***^ | 1.20 (1.08–1.33) ^***^ |
| Middle |  | na | 1.29 (1.15–1.44) ^***^ | 1.32 (1.17–1.48) ^***^ |
| Fourth |  | na | 1.48 (1.31–1.67) ^***^ | 1.52 (1.33–1.73) ^***^ |
| Richest |  | na | 1.66 (1.44–1.91) ^***^ | 1.68 (1.43–1.98) ^***^ |
| Place of residence |  |  |  |  |
| Rural |  | na | na | 1.00 |
| Urban |  | na | na | 0.96 (0.81–1.13) |
| Division of residence |  |  |  |  |
| Barisal |  | na | na | 1.00 |
| Chittagong |  | na | na | 0.93 (0.75–1.14) |
| Dhaka |  | na | na | 0.84 (0.68–1.04) |
| Khulna |  | na | na | 0.64 (0.52–0.80) ^***^ |
| Mymensingh |  | na | na | 0.94 (0.75–1.18) |
| Rajshahi |  | na | na | 0.62 (0.51–0.76) ^***^ |
| Rangpur |  | na | na | 0.58 (0.48–0.72) ^***^ |
| Sylhet |  | na | na | 1.24 (0.98–1.56) |
| Community level literacy |  |  |  |  |
| Low |  | na | na | 1.00 |
| Moderate |  | na | na | 1.22 (1.06–1.41) ^**^ |
| High |  | na | na | 1.39 (1.20–1.58) ^***^ |
| Community level poverty |  |  |  |  |
| High poverty |  | na | na | 1.00 |
| Moderate poverty |  | na | na | 1.06 (0.93–1.21) |
| Low poverty |  | na | na | 1.00 (0.82–1.23) |
| Middle or richest community |  | na | na | 1.13 (0.87–1.48) |
| Model statistics |  |  |  |  |
| ICC | 7% | 6.5% | 5% | 4.5% |
| AIC | 29762.94 | 24765.87 | 24556.75 | 24461.72 |
| BIC | 29781.98 | 24870.60 | 24718.62 | 24707.36 |

Note: ^**^ p<0.05, ^***^ p<0.01, na= Not applicable
